# The Human Mycobiome: Colonization, Composition and the Role in Health and Disease

**DOI:** 10.3390/jof8101046

**Published:** 2022-10-04

**Authors:** Paulina Belvoncikova, Petra Splichalova, Petra Videnska, Roman Gardlik

**Affiliations:** 1 Institute of Molecular Biomedicine, Faculty of Medicine, Comenius University, Sasinkova 4, 811 08 Bratislava, Slovakia; 2 Research Centre for Toxic Compounds in the Environment, Faculty of Science, Masaryk University, Kamenice 5, 625 00 Brno, Czech Republic

**Keywords:** fungi, gut mycobiome, oral mycobiome, skin mycobiome, genitourinary tract mycobiome, respiratory tract mycobiome, colonization, composition, dysbiosis

## Abstract

The mycobiome is the fungal component of the human microbial ecosystem that represents only a small part of this environment but plays an essential role in maintaining homeostasis. Colonization by fungi begins immediately after birth. The initial mycobiome is influenced by the gestational age of a newborn, birth weight, delivery method and feeding method. During a human’s life, the composition of the mycobiome is further influenced by a large number of endogenous and exogenous factors. The most important factors are diet, body weight, age, sex and antibiotic and antifungal therapy. The human mycobiome inhabits the oral cavity, gastrointestinal tract, respiratory tract, urogenital tract and skin. Its composition can influence the gut–brain axis through immune and non-immune mediated crosstalk systems. It also interacts with other commensals of the ecosystem through synergistic and antagonistic relationships. Moreover, colonization of the gut by opportunistic fungal pathogens in immunocompromised individuals can lead to clinically relevant disease states. Thus, the mycobiome represents an essential part of the microbiome associated with a variety of physiological and pathological processes. This review summarizes the current knowledge on the composition of the mycobiome in specific sites of the human body and its role in health and disease.

## 1. Introduction

The ecosystem represents a complex interconnected ecological community of living matter that forms the Earth’s biosphere, where organisms do not live separately, but rather in mutual relationships with each other. Likewise, a healthy human body is colonized by diverse microorganisms taxonomically belonging to all three cellular domains of life (Bacteria, Eukarya and Archaea), as well as non-cellular entities [1].

Fungi are the most abundant eukaryotes, with an estimated number of 2.2 to 3.8 million species, but it is supposed that only up to 8% are completely described [2]. In the past, they were considered as human pathogens and studied as individual species, not as a proportion of the human body’s ecosystem forming mutual relationships. Moreover, traditional cultivation methods could only provide information on morphology and viability but did not reflect an authentic composition of the fungal community due to the high percentage of non-culturable species [3,4]. However, the introduction of new high-throughput amplicon sequencing, shotgun metagenomic technologies and increasing knowledge about the importance of microorganisms as commensals in human health has turned the attention to all microbial members and their intra- and interdomain interactions.

Analogous to the terms bacteriome, archaeome or virome, the human mycobiome refers to the collective genome of all fungi (mycobiota when speaking of living matter itself), specifically unicellular yeasts and filamentous micromycetes, inhabiting our bodies [5,6]. The origin of the scientific phrase human mycobiome dates to 2010, when it was initially used in the article by Ghannoum et al. (2010). To our knowledge, this was the first study that described the oral mycobiome of healthy individuals and showed a significantly higher diversity of oral fungal commensals in comparison with standard culture-based methods [7]. Since then, the number of publications containing the keyword mycobiome has been increasing every year, and the published articles described the mycobiome not only in the oral cavity but also at the other body sites [8,9,10,11].

Although culture-based methods have been largely replaced by molecular-based high-throughput amplicon sequencing methods, the studying of the mycobiome still faces some technological limitations [12]. Different extraction methods can alter the bacterial community composition [12,13]. Among frequently used methods is the targeted sequencing of specific genes encoding ITS1, ITS2 and 18S rRNA regions [12,14]. Unfortunately, the primers designed to amplify these fungal rRNA genes show not only variable results, but also amplify genes of other eukaryotes. At the same time, none of the currently known primers can amplify the genes of all fungi. Moreover, eukaryotic rRNA operons have a variable ITS region length and number of tandem repetitions in different fungal species modifying the results of relative abundances [14,15]. In addition, the choice of reference database can play a role since some species do not have a united taxonomy of teleomorph and anamorph [16]. Thus, these limitations may lead to inconsistent results among studies performed by different research groups. Most of them agree at the phylum level; however, the major differences can be observed at the genus level. An extensive explanation of mycobiome methodology and its challenges are described in the review articles by Tiew and colleagues (2020) [17] and Cui and colleagues (2013) [18].

Up to now, the presence of the human mycobiome has been reported at five sites of the healthy human body, including the oral cavity, intestinal tract, respiratory tract, genitourinary tract and skin. However, it is not excluded that other mucosal surfaces are inhabited as well [7,8,11,19,20]. Naturally, the main focus is on the intestinal (gut) mycobiome, where the fungi play the important role in human health, immune system development and maintaining homeostasis [21,22,23,24]. Despite a high fungal diversity, the mycobiome takes only a minor part of the human microbial community. Its abundance varies across human body sites; for example, in the gastrointestinal tract, it covers less than 0.1%, whereas, on the skin, it takes up to 10% [24,25].

**Figure 1:**
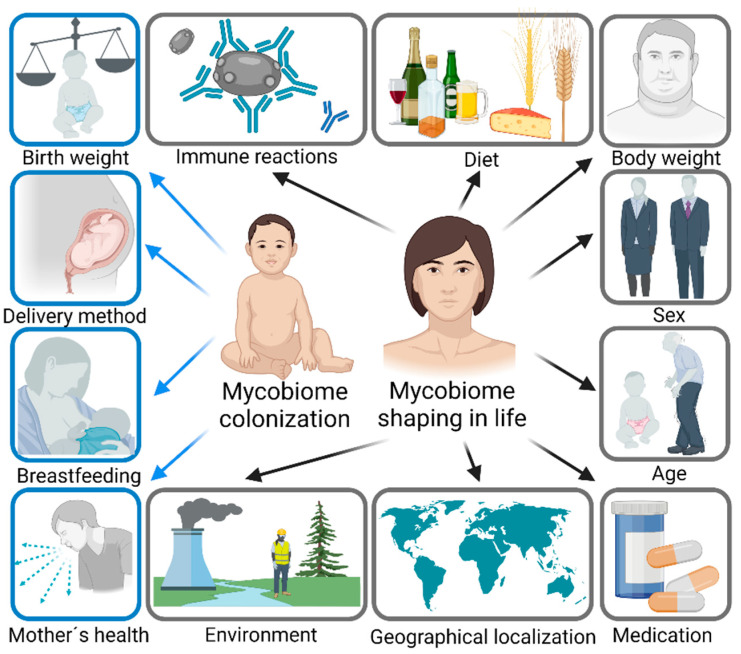
The main factors influencing the colonization and shaping the composition of the mycobiome in life. The colonization starts immediately after birth. Birth weight, related to gestational age, delivery method and breastfeeding vs. formula milk, as well as health and microbial colonization of relatives, can play a role. During life, the mycobiome is primarily shaped by diet, body weight, sex, age, medication and immune responses, but also the environment and geographical localization. Created with BioRender.com (accessed on 28 September 2022).

Contrary to the bacterial microbiome, which is relatively stable over time, the composition of the mycobiome varies substantially. Moreover, the human mycobiome is defined by a high interindividual and intraindividual variability [11]. The main factors influencing the development and composition of the mycobiome are described in Figure 1. The human mycobiome is shaped from birth and the source of fungal microorganisms can be either the mother (vertical transmission) or the environment (horizontal transmission) [26,27]. The gestational age, birth weight and delivery method can influence the development of the mycobiome [27,28]. Later on, the composition and the diversity of the mycobiome are largely modulated by the feeding method [26,27]. During adult life, the main factors influencing mycobiome composition are diet [29,30] and body weight [31,32,33]. Fungal microorganisms metabolize and simplify nutrient extraction, and help with digestion through enzyme and vitamin production [31,33,34]. Additional factors include the sex [31,35,36], age [36], antibiotic and antifungal use [37,38], lifestyle, hygiene and geography [19,39,40].

As part of the human body’s interconnected ecological community, they form interdomain and intradomain interactions with other colonizers. These interactions were described and summarized in a comprehensive review by Mishra et al. (2021) [41]. It seems that the cooperation of microorganisms could play a crucial role in health and disease. Forming mixed biofilms and the microbial co-habitation of bacteria and fungi could be implicated in, e.g., Crohn´s disease, cystic fibrosis and wound infections, as shown in vitro and in vivo [41].

The Mycobiome as a source of antigens is essential to the training of the immune system and its responses. Fungi activate fungus-specific pattern recognition receptors and immune mechanisms mediating defense against pathogens and tolerance towards helpful commensals [18]. However, there is limited knowledge about these host–fungal interactions in comparison with bacteriome studies [17].

Since the latest studies have described the human mycobiome as a minor but inherent component of the human microbial ecosystem, more efforts should be put into its analysis, with a focus on its role in health and disease. As the colonization of the human body starts along with birth, microorganisms accompany humans and shape their immunity from the very beginning. Modulating the immune system could be only one of many possible ways in which microorganisms influence us. Understanding the compositional changes in the gut mycobiome as well as its communication with other microbes can help us to understand its role in the pathogenesis of various disorders at body sites colonized by these microorganisms.

In the following sections of the article, we describe the mycobial colonization of five human body sites and the composition of their core mycobiome, as well as endogenous and exogenous factors known to influence the composition over life, with the focus on the diseases associated with disturbed mycobiome.

For this complex overview, databases Web of Science and PubMed were searched, using the keywords gut mycobiome, oral mycobiome, skin mycobiome, genitourinary tract mycobiome, respiratory tract mycobiome, colonization, composition and dysbiosis. No exclusion criteria on the date and type of article were applied; however, up-to-date articles were highly preferred. From the total number of searched results, we chose 167 clinical studies, experimental studies and reviews.

**Figure 2:**
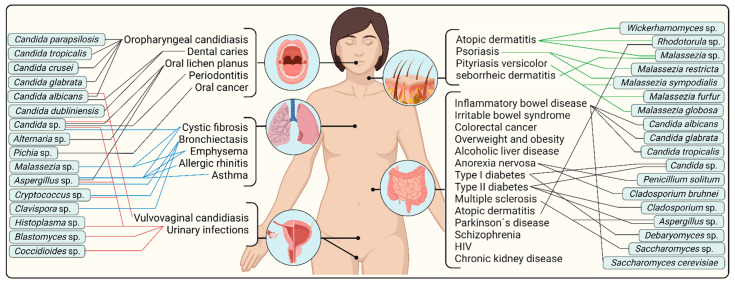
The summary of diseases in which gut mycobiome dysbiosis at various body sites could play a role. Fungal taxa potentially involved in the pathogenesis are included, specifically oral cavity mycobiome dysbiosis (black lines on the left), respiratory tract mycobiome dysbiosis (blue lines on the left), genitourinary tract mycobiome dysbiosis (red lines on the left), skin mycobiome dysbiosis (green lines on the right) and gastrointestinal mycobiome dysbiosis (black lines on the right). Created with BioRender.com (accessed on 28 September 2022).

Figure 2 shows a summary scheme of diseases in which the mycobiome could be implicated, as well as fungal taxa potentially involved in the pathogenesis. However, numerous previous studies have already discussed the perturbances in pathophysiologic states [17,18,41]. Therefore, herein, we also focus on the complex overview of a healthy human mycobiome, including less explored body parts to date. The summaries of the most common colonizers of the core mycobiome at various body sites are described extensively in tabular overviews. These provide a comprehensive overview of the most common fungal genera, the relative abundance of the genera or their frequency in clinical samples, the most common species and the list of all species that have been recently described at a particular body site. We believe that knowledge of these, as well as the initial colonization and dynamics of the mycobiome in a healthy person, are key to understanding compositional changes and mechanisms in the disease state.

## 2. Gut Mycobiome

The most studied human body site is the gut, colonized by both morphologic stages of microscopic fungi, yeasts and filamentous micromycetes. Gut colonizers facilitate the digestion of human-indigestible micronutrients and macronutrients, e.g., fiber and oligosaccharides. They produce a wide range of metabolites, such as short and medium chain fatty acids or gases arising from their fermentative metabolisms that could modulate the transition from yeast to a filamentous form (hyphae), oftentimes considered pathogenic, as reviewed in the publication by Sam and colleagues (2017) [42,43]. Moreover, the transport of these molecules could mediate an intercellular communication and be involved in the gut–brain axis, similarly to the bacterial microbiome [22]. In addition, the gut mycobiome plays an important role in the protection of the epithelial barrier against pathogens and stimulates immunity, e.g., through T-cell 17 immune mechanisms [44]. Thus, the gut mycobiome could be implicated in healthy mucosal homeostasis.

### 2.1. Composition of Gut Mycobiome

It is controversial to define a “core” gut mycobiome of the healthy individual due to high interindividual and intraindividual variability [11]. The presence of the mycobiome has been detected in the gut of at least 70% of healthy adults [45,46]. The most prevalent phylum is Ascomycota, covering 48% to 99% of all present species. Less abundant is the phylum Basidiomycota, with the relative abundance usually ranging from 0.5% to 14% of identified microscopic fungi, followed by the phylum Mucoromycota. It is assumed that these cover approximately 400 species of gut fungi [31,45,46]. A detailed list of chosen fungal genera is described in Table 1.

Across the phylum Ascomycota, numerous classes have been identified, namely *Saccharomycetes*, *Dothideomycetes*, *Sordariomycetes* and *Eurotiomycetes*. At the family level, *Saccharomycetaceae*, *Aspergillaceae*, *Cladosporiaceae*, *Debaryomycetaceae*, *Dipodascaceae* and *Pichiaceae* dominate, followed by less abundant families *Ceratocystidaceae*, *Hypocreaceae*, *Metschnikowiaceae*, *Nectriaceae*, *Thermoascaceae* or *Microascaceae* [30,33,45,60]. The most frequently described yeasts are genera *Candida* and *Saccharomyces* [11,31,49,51,52,53,54,55]. Various studies have described tens of other genera, e.g., yeasts *Debaryomyces*, *Meyerozyma*, *Toluraspora*, *Pichia*, *Clavispora*, *Cyberlidnera*, *Hanseniaspora*, *Geotrichum*, *Galactomyces* and *Zygosaccharomyces* [30,33,45,60]. When analyzing filamentous genera, the most common are *Paecilomyces*, *Cladosporium*, *Aspergillus* and *Penicillium* [11,31,52,54,55,58]. Some studies have also mentioned *Claviceps*, *Fonsecaea*, *Exophiala*, *Eurotium*, *Phialophora* and *Scopulariopsis* [30,31,33,45,61,62].

From the phylum Basidiomycota, most of the yeasts identified in the gut mycobiome belong to the classes *Malasseziomycetes*, *Tremellomycetes*, *Agaricomycetes*, *Microbotryomycetes* and *Cystobasidiomycetes* [31,33,45]. These are represented by families *Malasseziaceae*, *Cryptococcaceae*, *Corticiaceae*, *Sporidiobolaceae* and *Erythrobasidiaceae* [31,33,45]. The most commonly described genera are *Malassezia*, *Cryptococcus* and *Rhodotorula*, but *Filobasidium* and *Trichosporon* have also been reported [31,33,45].

The least abundant phylum Mucoromycota is represented by the class *Mucoromycetes*, family *Mucoraceae* and filamentous genera *Mucor* and *Rhizopus* [31,45].

Two mycotypes can be distinguished in the gut. Mycotype 1 is characterized by a high abundance of the genus *Saccharomyces*, but also other unclassified genera. On the contrary, Mycotype 2 preferably consists of the genera *Penicillium*, *Malassezia* and *Mucor* [55].

### 2.2. Gut Colonization and Shaping of Gut Mycobiome

The intestinal tract colonization of a newborn can take place both vertically (from mother to child) shortly after its birth or horizontally (from the environment to child) for a long period of time [27,63]. A common example of colonization impact could be a delivery mode. According to the study of European newborns, the earliest colonizers of C-section-born infants are the genus *Saccharomyces* and the class *Exobasidiomycetes*, whereas meconium samples of vaginally delivered children are colonized predominantly by the *Dothideomycetes* and *Pezizomycotina* [27]. Another method of colonization could be through drinking breast milk. The transition from maternal milk to a more diverse diet shifts its composition [26].

Over life, the diet is considered a crucial factor in modulating the gut mycobiome [34,64,65]. Food-borne fungi could be found in numerous food products: vegetables, fruit, fermented dairy products, meat and fermented beverages as shown in Table 2.

Some dietary components show a correlation even with the abundance of the specific genus or multiple species [64,65]. Genera *Saccharomyces* and *Hannaella* showed positive correlations with butter and animal fats, whereas the genus *Aspergillus* had a positive correlation with eggs and refined grain. On the contrary, *Saccharomyces* and *Aspergillus* correlated negatively with whole grain, but *Hannaella* correlated negatively with fish and shellfish [78]. Moreover, the genus *Fusarium* was identified in 88% of vegetarians, but only in 3% of omnivores [29,30]. A short-term switch to a strictly animal-based diet increased the relative abundance of the genus *Penicillium*, but decreased the genera *Debaryomyces* and *Candida* [64].Only approximately 20% of microscopic fungi colonizing the gut permanently inhabit this environment, e.g., the genus *Candida* and species *Geotrichum candidum* and *Rhodotorula mucilaginosa*. The rest, 80%, are considered allochthonous environmental and food-borne fungi, e.g., *Aspergillus* and *Penicillium* [30,45].

The gut mycobiome is related to lipid and carbohydrate metabolism through positive and negative correlations with various fungal taxa as shown in Table 3 and Table 4.

Lipid metabolic factors altering the gut mycobiome are the body mass index, body fat mass, fasting triglycerides, serum total cholesterol, low-density lipoprotein cholesterol and high-density lipoprotein cholesterol [33]. Carbohydrate metabolic parameters that could shape the gut mycobiome composition are fasting glycated hemoglobin, insulin and fasting glucose [33,43]. The gut mycobiomes of overweight and obese individuals differ from healthy eutrophic controls and have their specific composition [31,33]. Predominant genera of overweight patients are yeast *Candida* and *Pichia* and filamentous *Bipolaris*, *Beauveria*, *Exophiala*, *Syncephalastrum* and *Helminthosporium* [31]. On the contrary, in obese patients, genera *Candida*, *Nakaseomyces* and *Penicillium*, *Chaetomium* and *Emmonsia* dominate [31,33].

### 2.3. Gut Mycobiome Dysbiosis in Disease

It is important to distinguish what is and is not a healthy body state anymore and influence the onset of pathogenic processes. Intestinal dysbiosis leads to altered interactions among members of the microbiome and mycobiome, respectively, and these influence microbial penetration across the gut barrier [24].

A modified gut mycobiome composition was shown in numerous conditions: inflammatory bowel disease [81], irritable bowel syndrome [82], colorectal cancer [57], obesity [31,33], alcoholic liver disease [83], diabetes type I and II [79,84,85,86], multiple sclerosis [78,87], chronic kidney disease [88], atopic dermatitis [89], Parkinson’s disease [90], schizophrenia [91], etc. Moreover, correlations of fungal taxa with innate and adaptive immunity, e.g., effector CD4+ T cells and CD8+ T cells, memory CD4+ T cells, NK cells, regulatory B cells and other proinflammatory cytokines, were observed [78,92].

The gut mycobiome of patients diagnosed with inflammatory bowel disease displayed compositional differences compared to the control group; however, there were no disturbances between mycobiomes of Crohn´s disease and ulcerative colitis [56,81]. When investigating the most common gut colonizers, an increased abundance of *C. albicans* [81], *C. glabrata* and *C. tropicalis* was observed in the patients with IBD [93,94,95]. Additionally, the relative abundance of *C. albicans* significantly correlated with the remission and relapse stages of inflammatory bowel disease, while another species, *C. tropicalis*, displayed interactions with anti-*Saccharomyces cerevisiae* antibodies that are known as biomarkers associated with Crohn´s disease [81,94]. However, the proportion of the species *Saccharomyces cerevisiae* appeared controversial [81,96]. Whereas Sokol et al. (2017) described a decrease in the relative abundance of this species, Lewis et al. (2015) reported its increase in Crohn´s disease [96].

A considerable biodiversity decrease was illustrated in overweight and obese individuals in contrast to healthy eutrophic controls, respectively. A comprehensive analysis of young anorexic woman exposed unique mycobiome species *Penicillium solitum* and *Cladosporium bruhnei* [32]. Nevertheless, a study of 59 anorexia nervosa patients mentioned no changes in alpha diversity [97].

Modulating the gut microbial colonizers through dietary components could have an anti-diabetic activity [98]. The gut mycobiome in type II diabetes displayed compositional differences at the phylum level, as well as at the genus level, compared to healthy individuals [84,85]. An increase in the relative abundance of *Candida*, *Meyerozyma*, *Aspergillus* and *Cladosporium* was shown. In contrast, 21 genera were significantly reduced in patients, e.g., *Saccharomyces*, *Trichoderma*, *Clavispora*, *Rhizopus* and *Wickerhamomyces* [84,85]. The bacterial and fungal dysbiosis in type I diabetic children with beta-cell autoimmunity was in association with intestinal inflammation [86]. An increased relative abundance of the genus *Debaryomyces* and decreased abundance of *Malassezia* were disclosed [86]. An increased abundance of the genus *Candida* was reported in diabetic patients [79,99]. This increased abundance of saccharide-metabolizing yeast could probably be a consequence of elevated blood glucose levels or modified immunological responses.

The gut mycobiome of multiple sclerosis patients had a higher alpha diversity, interindividual variability and over-representation of genera *Saccharomyces* and *Aspergillus* compared to a healthy control group [78]. Despite that, it preserved its stability for a 6-month period, suggesting that common species are permanent colonizers over time. Gut mycobiome enrichment and numerous correlations with bacterial taxa were reported in people with HIV [92]. The overgrowth of the genus *Rhodotorula* was related to atopic dermatitis manifestation in infants [89].

Provided data suggest that fungal dysbiosis may contribute to the pathogenesis of numerous intestinal and extraintestinal disorders. Knowledge of the intradomain and interdomain interaction of fungal and bacterial colonizers in gut mycobiome dysbiosis could lead to the development of novel therapeutic strategies in the future.

## 3. Oral Mycobiome

The oral microbiome is one of the most complex and diverse microbial ecosystems in the human body [100]. While eating, foodborne microscopic fungi (or their conidia) pass through our digestive system. Starting from the oral cavity, the oral mycobiome has been relatively well described [7]. The inter-kingdom interactions between bacteria and fungi are essential in the oral cavity and contribute to oral health and the establishment of distal gastrointestinal tract functions [100,101,102]. These interactions are crucial for maintaining the balance between health and disease, not only locally but also systematically [103]. Highly abundant genera, such as *Candida*, are associated with a distinct oral ecology of a decreased pH. Some of these fungal species can serve as the main etiological agent of oral mucosal mycosis. However, the role of other metabolically active colonizers, such as the lipid-dependent *Malassezia*, is still unclear. Less abundant oral mycobiome species could potentially serve as opportunistic pathogens in immunocompromised hosts [104].

### 3.1. Composition of Oral Mycobiome

The composition of the oral mycobiome is stable, yet it shows a high interindividual variability [100]. The majority of the oral adult mycobiome composition represents the phylum Ascomycota, Basidiomycota, Glomeromycota and Mucoromycota. These four groups include approximately 81 genera and 101 species of fungi [7,105]. A detailed list of chosen fungal genera is described in Table 5.

The most common representatives of the basal oral mycobiome originate from the phylum Ascomycota, namely the yeast-like genus *Candida* [7,61,101,109,110]. Less prevalent yeasts that have been reported are *Aureobasidium*, *Saccharomyces* and *Pichia* [7,61,100,107,108]. Some of the identified filamentous micromycetes are genera *Penicillium*, *Cladosporium*, *Aspergillus* and *Alternaria* [7,61,100,101,105,106,107,108]. Some studies reported airborne fungi, genera *Phoma* and *Epicoccum*, whose spores could be inhaled by the study participants without the subsequent colonization of the oral cavity [7,61,106].

Basidiomycota representatives include *Rhodotorula*, *Malassezia* and *Cryptococcus*. Minor relative abundances of other representatives are rarely reported [7,61,105,106].

### 3.2. Oral Cavity Colonization and Shaping of Oral Mycobiome

The colonization of the oral cavity by fungi starts during childbirth [103] and progresses with age [102]. The abundance of *Candida*, one of the most abundant genera of the oral cavity, is age-dependent. Its relative abundance is lower in infants compared to one-year-old children [111]. The mycobiome composition is similar in children born by either vaginal or C-section delivery, but the relative abundance of certain species, such as *C. orthopsilosis*, is significantly higher in section-born infants [112,113]. The fungal colonization is higher in neonates born vaginally than in those born by C-section. Late colonization may also occur due to longer stays in the neonatal intensive care unit [103]. The very low birth weight neonates were more frequently colonized by non-albicans *Candida* sp. than neonates with a birth weight above 1500 g, who were mostly colonized by *C. albicans* [103]. Children’s oral cavity contains over 40 fungal species with the dominant species *C. albicans* [40]. Most studies suggest that the type of feeding does not influence the oral mycobiome composition [102,103]. *Candida* species abundance is the same for infants who are breastfed, bottle-fed or those who are on both patterns of feeding [114]. *Candida* species can also contribute to dental caries development [40].

The significant associations of the oral mycobiome composition with an increased age, teeth loss [105], low salivary flow rates and presence of dental caries [40,112], but also diet, personal oral hygiene, stress and drug exposure [115], were observed. Geographical differences were also described, e.g., a lower oral *Candida* abundance in healthy people in Asia compared with Western countries [116]. In addition, *C. glabrata* and *C. dubliniensis* were detected in the oral mycobiome of people with a low BMI [116].

### 3.3. Oral Mycobiome Dysbiosis in Disease

The dysbiosis of the oral mycobiome influences interactions between fungi and bacteria and might be associated with numerous conditions, e.g., dental caries [117], oropharyngeal candidiasis [118], oral lichen planus [101], periodontitis [105] and others.

Numerous studies have suggested a possible role of the genus *Candida*, the common isolate, in oral dysbiosis. This genus can participate in biofilm structure and function. It is able to form dentinal tubules and bind to denatured collagen and secrete aspartyl proteases, resulting in the demineralization and dissolution of the dental hard tissues [40]. The abundance of species *C. albicans* and *C. dubliniensis* correlated with dental caries in early childhood, indicating that *C. dubliniensis* could be implicated in disease pathogenesis [117]. A higher abundance of *C. glabrata* resulted in tongue infections and the interactions between *C. glabrata* and hyphae of *C. albicans* established oropharyngeal candidiasis [118]. Other etiological agents of oropharyngeal candidiasis were *C. parapsilosis*, *C. tropicalis* and *C. crusei* [118]. Fungal dysbiosis was also associated with the oral lichen planus, the chronic oral mucosa disease. Significantly higher abundances of genera *Candida* and *Aspergillus* in patients with reticular oral lichen planus and of *Alternaria* and *Sclerotiniaceae* in patients with erosive oral lichen planus were observed [101]. On the contrary, no compositional changes in the overall mycobiome diversity were shown in patients with periodontal disease compared to healthy study individuals [105]. Peters et al. (2017) manifested that the abundance of the genus *Candida* was increased in patients, but not significantly [105].

A recent study proposed that compositional alterations of the oral mycobiome caused by smoking tobacco could be implicated in oral cancer by increasing the abundance of pathogenic fungi that promote opportunistic infections [119]. A decreased oral fungal diversity and increase in the genus *Pichia* correlated with the severity of the oral lesions.

## 4. Skin Mycobiome

The skin is the first barrier between the environment and the human body, protecting against potential pathogens [120]. However, it is colonized by numerous commensal microorganisms, maintaining homeostasis through the production of antimicrobial peptides and protection against external harmful microorganisms, and mediating lipid and urea degradation by lipase and urease enzymes secretion [121,122]. These commensals contribute to immune response development and are involved in potential microbial communication with bacteria, modulating their physiology and virulence [123].

### 4.1. Composition of Skin Mycobiome

The human skin fungal ecosystem represents less than 10% of all skin microbes [9]. The skin mycobiome consists of at least 168 genera, taxonomically originating from the phyla Basidiomycota and Ascomycota [19,39,124], and these are body-site-specific [39]. Parts of the body in pairs, such as forearms and palms, are colonized by the same dominant taxa [39]. A detailed list of chosen fungal genera is described in Table 6.

The most common colonizer of human skin is the yeast of the genus *Malassezia*. It dominates several parts of the body, such as the inner side of the elbows, back, outer auditory canal, nostril and various facial areas. Within each observed sample, it represents more than 57% of the microscopic community on average [19,124].

From the Basidiomycota, the skin of healthy people is colonized by the genus *Cryptococcus* [19,39]. Rarely identified are other genera, e.g., *Aspergillus*, *Penicillium* and *Epicoccum* [19,123].

### 4.2. Skin Colonization and Shaping of Skin Mycobiome

The colonization of the skin by fungi starts during childbirth and is characterized by a high inter-individual variability [27,113,120]. Children born vaginally most likely acquire microscopic fungi from the mother´s vagina, whereas children born via C-section acquire fungi primarily found on the mother´s skin [27,113]. The relative abundance of specific taxa, including *Saccharomyces cerevisiae*, *Candida tropicalis*, *C. parapsilosis*, *C. albicans* and *C. orthopsilosis*, was influenced by the delivery mode in the study of American infants [113]. No alpha diversity differences were recorded during the first month of life between vaginal and C-section delivery. However, only vaginally derived infants showed an alpha diversity increase over the first month of life [113]. On the contrary, a study of Chinese infants proposed no significant change in the mycobiome composition within the first six months of life [120].

The skin mycobiome changes and develops compositionally from childhood to adulthood, with a profound shift in community composition during puberty. The most common species of pre-puberty children were *Aspergillus*, *Epicoccum* and *Phoma*. The dominant species of *Malassezia* before puberty was *M. globosa*. On the contrary, individuals in the post-pubertal age were predominantly colonized by *M. restricta*. Gender also affected the composition of the skin mycobiome. *Epicoccum* and *Cryptococcus* were more abundant in adolescent boys, whereas, in girls, it was the genus *Malassezia* [36].

During life, the skin mycobiome could vary across body site localization or hygiene as shown by culture-dependent and independent methods [19,128,129,130]. Sebaceous body sites (e.g., face, chest and back) had a higher fungal diversity of lipophilic species compared to moist or dry skin sites [120,128]. Site specification is typical for *Malassezia*: the higher abundance of *M. globosa* on the scalp and forehead and lower abundance on the back were observed, while the relative abundance of *M. sympodialis* showed an inverse pattern [123]. *Malassezia arunalokei* was more abundant on the forehead and cheek compared to the scalp [129]. Seasonal and gender-related changes in the skin mycobiome were observed [131,132].

### 4.3. Skin Mycobiome Dysbiosis in Disease

The skin mycobiome comprises harmless commensals; however, it also comprises opportunistic pathogens, whose overgrowth leads to morphological changes from budding yeasts to a pseudohyphal form invading the stratum corneum layer and promoting skin infections [133]. Inter-kingdom and inter-species microbial interactions can worsen the disease or change relationships from opportunistic to pathogenic [131,134,135]. Fungal dysbiosis could be associated with numerous skin diseases often present in a site-specific manner, including atopic dermatitis in the arm and leg creases [136], psoriasis on the elbows and knees [132], pityriasis versicolor [137], seborrheic dermatitis [138], etc. The prevalence of fungal infections on the skin, hair and nails varies from 20 to 25% worldwide [139].

The dysbiosis of the major commensal skin fungi, *Malassezia* sp., is associated with numerous skin disorders [123,137]. The species *Malassezia globosa* colonized antecubital flexures at the neck of healthy individuals but was absent in patients with atopic dermatitis [136]. Similarly, a higher relative abundance of *M. globosa* in controls was reported by another study [140]. This growth restriction could be a consequence of dry skin and insufficient growth conditions usually provided by the bacterium *Cutibacterium acnes*, which were decreased in patients with atopic dermatitis as well [136]. Furthermore, *Malassezia restricta* and *Malassezia sympodialis* were significantly associated with psoriasis at the back and elbow body sites as shown by LEfSe analysis [135]. In addition, *Malassezia globosa* has been considered a common etiological agent of Pityriasis versicolor (also known as tinea versicolor), present as hyperpigmented and hypopigmented skin lesions [133,141,142]. Nevertheless, species *Malassezia furfur* and *Malassezia sympodialis* have been isolated from the clinical isolates as well [137]. Lastly, an increased relative abundance of the genus Malassezia and decreased fungal diversity were reported in the study of patients with facial seborrheic dermatitis [38]. These were modified by ketoconazole treatment.

Besides the genus *Malassezia*, the perturbances of the skin mycobiome have been shown; for instance, in the study of nine 12-month-old infants with atopic dermatitis. Specifically, genera *Rhodotorula*, *Wickerhamomyces*, *Kodamaea*, *Acremonium* and *Rhizopus* showed the highest rates of perturbance [89].

## 5. Genitourinary Tract Mycobiome

Fungi are important members of the vaginal ecosystem in healthy women and the vaginal mycobiome was first described by Castellani in 1929 [8,143]. *Candida albicans*, an opportunistic fungal pathogen, colonizes 20% of women without causing any overt symptoms, yet is one of the leading causes of infectious vaginitis. Understanding its mechanisms of commensalism and pathogenesis is essential to developing more effective therapies [144]. In contrast, before the advent of culture-independent analyses, the uninfected urinary tract had been assumed to be a sterile environment [145].

### 5.1. Composition of Genitourinary Mycobiome

The vaginal mycobiome consists primarily of yeast-like microorganisms originating from Ascomycota and Basidiomycota [8,143]. The research of the team Guo et al. (2012) [146] mentions peronosporomycota (oomycota) as well, but these have not been reported in other studies so far. At least 22 genera of microscopic fungi naturally inhabit the vaginal microenvironment of women [144]. A detailed list of chosen fungal genera is described in Table 7.

The vast majority of genera belong to Ascomycota and order *Saccharomycetales*, similarly to the oral cavity and gut mycobiome [8]. The most ubiquitous natural colonizer, which is a part of the vaginal mycobiome of almost every healthy woman, is the genus *Candida* and, namely, the species *C. albicans* [8,148]. The second most common species is *C. glabrata* [148,149], but numerous other species have been identified in the vaginal mycobiome so far, e.g., *C. krusei*, *C. tropicalis*, *C. parapsilosis*, *C. dubliniensis* and *C. guillermondi* [8,146,149]. Another representative commonly occurring in the vaginal mycobiome is the species *S. cerevisiae* [149].

Other taxa from the Ascomycota of yeasts and filamentous fungi that have been identified are from orders *Capnodiales*, *Eurotiales*, *Pleosporales* and *Helotiales*, namely the species *Cladosporium perangustum*, *Eurotium amslodami* and *Alternaria alternata* [8]. From the Basidiomycota, the yeast-like genus *Rhodotorula* has been reported [8].

The mycobiome of the urinary tract has not been clarified at present. However, one study described genera *Candida*, *Saccharomyces* and *Malassezia* as potential colonizers [145].

### 5.2. Genitourinary Tract Colonization and Shaping of Genitourinary Mycobiome

Within weeks after birth, fungi colonize various body sites [150]. A chronic colonization of the urogenital tract with fungi is common, especially for *Candida* spp. The carriage rate of *Candida* among healthy adults ranges from 30 to 70% [126].

The source of the vaginal mycobiome is a dietary intake, environmental contacts and living habits [146]. Similarly to other body sites, the composition of the initial vaginal mycobiome is influenced by the type of delivery, as vaginally delivered children possess a mycobiome originating from their mother´s vagina, whereas the children delivered by C-section obtain mycobiome from the mother’s skin and environment [150,151]. The vaginal mycobiome is different in women with a menstruation cycle, and in pre-adolescent, post-climacteric or pregnant women. The composition changes with the vaginal epithelium structure and depends on the level of sex hormones [150].

The composition of the urinary tract mycobiome is influenced by a multitude of factors, including age, gender, environmental variations and hygiene [145].

### 5.3. Genitourinary Mycobiome Dysbiosis in Disease

The vaginal microbiome and mycobiome are closely associated with the development of reproductive disorders. The dysbiosis of the vaginal microenvironment, together with immune defects and an infraction of epithelial integrity, leads to vulvovaginal candidiasis, one of the most common forms of infectious vaginitis caused by *C. albicans* [144]. The dysbiosis of *Lactobacillus* spp. and other lactic-acid bacteria, producing short chain fatty acids and lactate, probably allows for the switch of this dimorphic fungus from budding yeast to a virulent pseudohyphal form [144,152,153].

The study of intrauterine adhesion patients described *Candida*-related differences in the gut mycobiome composition and numerous fungal–bacterial correlations in cervical canal and middle vagina sites [154]. There are multiple studies describing the role of *Candida* strains in the context of vaginal pathophysiology, but there are no studies describing the comprehensive impact of vaginal mycobiome dysbiosis on women´s health. However, some studies hypothesized that vaginal microbial dysbiosis could contribute to human papillomavirus infection and carcinogenesis [155].

Fungal urinary infections are usually caused by the dysbiosis of *Candida* spp., but also the common invasive fungal species, including *Cryptococcus*, *Aspergillus*, *Mucoraceae*, *Histoplasma*, *Blastomyces* and *Coccidioides*. Fungal urinary tract infections are life-threatening due to the limitations in culture methodologies, frequent low suspicion of fungal involvement and the lack of preventative and therapeutic options [145]. As the urinary tract was considered sterile until recently and only a very limited number of articles have been devoted to the urinary tract mycobiome, it is not possible to deduce conclusions regarding the dysbiosis of this body site.

## 6. Respiratory Tract Mycobiome

Until recently, it was thought that the lungs of healthy people are sterile. However, it was found that the respiratory tract, including the lungs, have diverse microbiota influencing the pathogenesis of different diseases [156,157]. The lung mycobiome has its composition and evolution, which is unique and specific to each individual [158]. In healthy people, fungal spores, which are parts of the environment, are effectively removed from the respiratory tract through mucociliary clearance and phagocytosis [159,160]. In contrast, in immunosuppressed patients and in patients with a defect such as neutropenia, asthma or cavitating lung disease, fungal spores can persist and colonize, leading to the development of disease [160,161].

### 6.1. Composition of Respiratory Mycobiome

The number of studies describing respiratory mycobiome is limited. Despite that, already published literature describes mostly Ascomycota and Basidiomycota phyla at various sections of the respiratory tract, as well as those obtained by various sampling methods [20,110,159]. A detailed list of chosen fungal genera is described in Table 8.

Through the sequencing of the exhaled breath condensate samples, genera *Cladosporium*, *Aspergillus* and *Alternaria* and numerous *Penicillium* species were described [159]. On the contrary, analysis of the bronchoalveolar lavage samples revealed yeast-like fungi *Saccharomyces cerevisiae*, *Candida*, *Meyerozyma guilliermondii (Candida guilliermondii)*, *Pichia jadini* and *Debaryomyces* and filamentous *Cladosporium*, *Aspergillus* and *Penicillium* [20,110,161]. However, the abundance of some of these taxa could have been a result of the lung transplantation that patients underwent [110].

The induced sputum examination showed *C. albicans*, *C. dubliniensis* and *Aspergillus*. From the Basidiomycota, *Cryptococcus magnus* was identified, but also numerous other species, such as *Peniophora incarnata*, *Peniophora cinerea*, *Daedaleopsis confragosa*, *Sistotrema brinkmannii* and *Stereum hirsutum*, that occur in nature in connection with plants [20]. We assume that these species could get into the airways with microaspiration and are not permanent colonizers of the respiratory tract.

### 6.2. Respiratory Tract Colonization and Shaping of Respiratory Mycobiome

In the past, studies on respiratory tract colonization focused mostly on microorganisms in the context of pathogens that could potentially trigger diseases in humans [163,164]. Due to this, respiratory tract mycobiome studies have been lacking. Up to 2015, it was not clear whether there is a respiratory tract mycobiome in immunocompetent individuals without the pathogenic condition or not [20]. Some studies indicated that the presence of eukaryotic microorganisms in the respiratory tract is transient since the abundance of these species fluctuates over time [164]. The entry of these species into the airways can be a result of micro-aspiration from the oral cavity [100]. Other studies proposed that, although the composition of the mycobiome from individual airway sections overlaps, these sections also contain genera specific to only one of them. In addition, the respiratory mycobiome also shows differences compared to oral cavity mycobiome [20]. From these results, we suppose that the respiratory tract can be colonized by fungi permanently.

The respiratory tract mycobiome varies throughout parts of the respiratory system as shown above in the composition chapter [157]. It is also influenced by the composition of other mycobiomes. The intestinal microbiome influences the oropharyngeal mycobiome, which may influence the respiratory mycobiome and host immune response through micro-aspiration [100,158]. Other factors influencing the respiratory tract mycobiome are geographic variability (climate, humidity and air quality), genetics, lifestyle and dietary differences [17].

### 6.3. Respiratory Tract Mycobiome Dysbiosis in Disease

Mycobiome dysbiosis contributes to respiratory disease pathogenesis. Characteristic mycobiome profiles exist in chronic respiratory diseases [17]. The increased abundance of *Malassezia*, *Candida* and *Aspergillus* in patients with cystic fibrosis was observed [17,158,165]. In addition, a shift in the Basidiomycota:Ascomycota ratio was observed [161,165]. A higher abundance of *Aspergillus*, *Cryptococcus* and *Clavispora* was observed in patients with bronchiectasis [17]. *Aspergillus* and *Malassezia* are associated with combined pulmonary fibrosis and emphysema.

A human invariably inhales fungal spores, which are potential allergens [166]. *Aspergillus* spores can be implicated in the airway’s allergic disease production, such as allergic rhinitis and asthma [24]. An increased chance of asthma and allergies is also influenced by antibiotics use, which allows for the overgrowth of commensal fungi [24,167]. The most frequent species in the airways of asthma patients is *Malassezia pachydermatis*, the species associated with atopic dermatitis [166]. In addition to this, an increased abundance of *Psathyrella*, *Malassezia*, *Termitomyces* and *Grifola* was observed [17]. However, the effect of respiratory tract mycobiome dysbiosis on systemic health is currently not known.

## 7. Conclusions

Despite constituting a small proportion of the whole microbial ecosystem, the mycobiome plays an essential role in maintaining host homeostasis. Gut and oral mycobiomes have already been well-described, but studies on the skin, genitourinary and respiratory tract mycobiomes are lacking. Many already published papers have focused either on specific yeasts and cultivation methods or have attributed the presence of fungi to infection and not to the natural microflora. Therefore, we believe that the added value of this article is a complex tabular overview of a healthy mycobiome of less-explored body sites provided on the basis of recent literature. Knowledge of the natural fungal flora would help us to understand the changes in dysbiosis.

One possible explanation for why mycobiome research is still falling behind the bacterial microbiome could be that the research methodology is not fully standardized yet compared with the 16S rRNA microbiome analysis, and the fungi represent a minor part of the microbial composition, increasing the risk of contamination. Moreover, only limited information is known about its interactions with other commensals at the moment. Therefore, more efforts should be put into analyzing not only its variable composition but also the intercommunication with other microorganisms and released microbial metabolites, interactions with the host organism and their complex impact on human health. We believe that these could be potential studies in future research. Knowledge of these could provide a comprehensive point of view, helping to enlighten the pathogenesis of various disorders at body sites colonized by these microorganisms.

## Figures and Tables

**Table 1:** The list of chosen gut mycobiome colonizers. The table summarizes the relative abundance (or frequency) of representative taxa, number and summary of all species described in the studies.

Genus	The Most Common Species	Abundance/ Frequency	Number of Species	List of all Species	References
*Saccharomyces*	*S. cerevisiae*	~100% [47]; 96.8%[11]; 23% [48]; 12.80% [49]; 6.94% [31]; 5.1% [50]; 1.14% [35]	5	*S. cerevisiae*, *S. paradoxus*, *S. caariocanus*, *S. bayanus*, *S. castellii*	[11,31,35,47,48,49,50,51,52,53,54,55,56,57]
*Candida*	*Candida* sp.	59.72% [31]; 2.48% [49]	21	*C. albicans*, *C. tropicalis*, *C. dubliniensis*, *C. glabrata*, *C. krissi*, *C. rugosa*, *C. austromarina*, *C. parapsilosis*, *C. lusitaniae*, *C. pararugosa*, *C. deformans*, *C. fermentati*, *C. intermedia*, *C. metapsilosis*, *C. zeylanoides*, *C. sake*, *C. krusei*, *C. kefyr*, *C. guilliermondii*, *C. lambica*, *C. humilis*	[11,29,31,32,35,47,48,49,50,51,52,53,54,55,56,58,59]
	*C. albicans*	80.8%[11]; 62.7% [50]; 39.8% [35]; ~30% [47]; 28% [48]; 16.67% [31]; 11.16% [49]; 5.81% [56]			
*Malassezia*	*M. restricta*	88.30% [11]	5	*M. slooffiae*, *M. globosa*, *M. pachydermatis*, *M. restricta*, *M. sympodialis*	[29,32,47,49,51,54,58]
*Penicillium*	*Penicillium* sp.	65.28% [31]; 7% [11]	14	*P. turbatum*, *P. chrysogenum*, *P. freii*, *P. camemberti*, *P. brevicompactum*, *P. allii*, *P. dipodomyicola*, *P. sacculum*, *P. italicum*, *P. glabrum*, *P. verrucosum*, *P. crutosum*, *P. paneum*, *P. roqueforti*	[11,29,31,32,35,49,52,54,55,58]
	*P. brevicompactum*	3.72% [35]			
*Aspergillus*	*Aspergillus* sp.	58.33% [31]	16	*A. cristasus*, *A. flavipes*, *A. glaucus*, *A. pseudoglaucus*, *A. oryzeae*, *A. flavus*, *A. fumigatus*, *A.* cf. *niger*, *A.* cf. *carbonarius*, *A.* cf. *foetidus*, *A.* cf. *tubingensis*, *A. restrictus*, *A. versicolor*, *A. clavatus*, *A. ochraceoroseus*, *A. rambelli*	[11,29,31,32,35,47,49,54,55,57,58]
	*A. versicolor*	4.95% [49]			
*Galactomyces*	*G. candidum*	46.1%[11]; 6.98% [56]; 0.83% [49]	2	*G. geotrichum*, *G. candidum*	[11,32,49,51,56,58]
*Trichosporon*	*Trichosporon* sp.	30.56% [31]	4	*T. asahii*, *T. faecale*, *T. caseorum*, *T. cutaneum*	[31,32,35,51,52,59]
	*T. asahii*	0.57% [35]			
*Rhodotorula*	*Rhodotorula* sp.	8.50% [50]	4	*R. mucilaginosa*, *R. rubra*, *R. graminis*, *R. glutinis*	[31,32,35,50,54,55,56]
	*R. mucilaginosa*	15.28% [31]; 12.6% [35]; 6.98% [56]			
*Geotrichum*	*G. silvicola*	9.72% [31]	1	*G. silvicola*	[31,32,50,51,59]
*Cryptococcus*	*C. carnescens*	4.65% [56]	6	*C. amylolyticus*, *C. carnescens*, *C. neoformans*, *C. saitoi*, *C. albidus*, *C. luteolus*	[29,35,47,50,56]
*Pichia*	*P. manshurica*	3.43% [35]; 1.39% [31]	5	*P. kudriavzevii*, *P. fermentans*, *P. manshurica*, *P. kluyveri*, *P. caribica*	[11,29,31,35,54]
*Exophiala*	*E. dermatitidis*	1.39% [31]	4	*E. mesophila*, *E. heteromorpha*, *E. equina*, *E. dermatitidis*	[29,31,32,57]
*Cladosporium*	*Cladosporium* sp.	1.16% [56]	1	*C. cladosporioides*	[11,54,55,56,58]
	*C. cladosporioides*	not found			
*Mucor*	*M. circinelloides*	0.57% [35]	2	*M. circinelloides*, *M. racemosus*	[31,35,53,54]
*Alternaria*	*A. alternata*	not found	7	*A. alternata*, *A.* cf. *arborescens*, *A* cf. *brassicola*, *A.* cf. *citri*, *A.cf. mali*, *A.* cf. *tenuissima*, *A. metachromatica*	[4,11,29,32,48,55]
*Debaryomyces*	*D.fabryi*	not found	3	*D. hansenii*, *D. fabryi*, *D. prosopidis*	[11,32,52,54,55,57]

**Table 2:** The list of food products colonized or contaminated by food-borne microscopic fungi.

Food Product	Microscopic Fungi	References
**Fruit and vegetables**		
Fresh citrus and grape	*Candida prunicola*, *Pichia fermentans*	[66]
Fresh apple, plum and pear	*Saccharomyces cerevisiae*, *Pichia kluyveri*, *Pichia kudriavzevii*, *Galactomyces candidus*, *Hanseniaspora uvarum*, *Hanseniaspora guilliermondii*	[67]
Peeled fruit salads	*Candida* sp., *Debaryomyces* sp., *Rhodotorula* sp., *Penicillium* sp., *Cladosporium* sp.	[68]
Dried fruit	*Cladosporium* sp., *Aspergillus niger*, *Aspergillus tubingiensis*, *Penicillium palitans*	[68,69]
Various fresh vegetables (salad, tomato, cucumber, green inion, lettuce, spinach, etc.)	*Geotrichum* sp., *Alternaria* sp., *Cladosporium* sp., *Penicillium* sp.	[68,70]
**Dairy**		
Various cheeses (Blue cheese, Camembert, Cheddar)	*Penicillium* sp., *Candida* sp., *Scopulariopsis* sp.	[64]
Acidophilus milk	*Saccharomyces fragilis*, *Candida pseudotropicalis*	[71,72]
**Meat**		
Various meats (fermented sausage, dried meat, salami, ham)	*Debaryomyces* sp., *Penicillium* sp.	[64]
**Beverages**		
Wine	*Hanseniaspora* sp., *Saccharomyces* sp.	[73,74]
Beer	*Brettanomyces* sp., *Saccharomyces* sp.	[73,74]
Sake	*Aspergillus* sp., *Saccharomyces* sp.	[73]
**Other**		
Various nuts (pecan, almond, walnut, pine nut)	*Aspergillus* sp. *Penicillium* sp., *Alternaria* sp., *Cladosporium* sp., *Rhizopus* sp., *Fusarium* sp.	[69]
Koji	*Aspergillus* sp., *Rhizopus* sp.	[75]
Soy sauce	*Aspergillus* sp., *Hansenula* sp., *Zygosaccharomyces* sp.	[73]
Steamed pastry	*Wickerhamomyces anomalus*	[76,77]

**Table 3:** The list of negative and positive correlations between lipid metabolism indices and gut mycobiome taxa.

Lipid Metabolism					
	BMI Index and Fat Mass	Serum Total Cholesterol Fasting Triglycerides	LDL ^1^ Cholesterol	HDL ^2^ Cholesterol	References
**Negative** **correlations**	*Agaricomycetes*	*Eurotiomycetes*	*Mucoraceae*	*Sacharomycetes*	[33,79]
	*Nectriaceae*	*Hypocraceae*		*Tremellomycetes*	
	*Mucoraceae*	*Mucoraceae*	*Mucor* sp.	*Cystobasidiomycetes*	
	*Mucor* sp.	*Mucor* sp.	*Candida* sp.	*Erythrobasidiaceae*	
	*Penicillium* sp.	*Candida* sp.			
**Positive** **correlations**	*Sacharomycetes*	*Dipodascaceae*	*_*	*Aspergillaceae*	[33,65,78].
	*Tremellomycetes*			*Penicillium* sp.	
	*Cystobasidiomycetes*			*Eurotiomycetes*	
	*Erythrobasidiaceae*			*Candida dubliniensis*	
	*Dipodascaceae*				
	*Aspergillus* sp.				
	*Hannaella* sp.				

^1^ Low-density lipoprotein cholesterol. ^2^ High-density lipoprotein cholesterol.

**Table 4:** The list of negative and positive correlations between carbohydrate metabolism indices and gut mycobiome taxa.

Carbohydrate Metabolism					
	Glucose	Insulin	Glycated Haemoglobin		References
**Negative** **correlations**	*Candida* sp. *C. dubliniensis*	_	*Agaricomycetes*	*Mucor* sp.	[33,80]
			*Ceratocystidaceae*	*Monilliela* sp.	
			*Debariomycetaceae*	*Eupenicillium* sp.	
			*Mucoraceae*		
			*Corticiaceae*	*Ceratocystis* sp.	
**Positive** **correlations**	*Eurotium* sp.	*Ascomycota*	*Eurotium* sp.	-	[33]

**Table 5:** The list of chosen oral mycobiome colonizers. The table summarizes the relative abundance (or frequency) of representative taxa, number and summary of all species described in the studies.

Genus	The Most Common Species	Abundance/ Frequency	Number of Species	List of All Species	References
*Candida*	*Candida* sp.	100% [106]; 100% [105]; 85% [101]; 75% [7]; 67% [100]	6	*C. albicans*, *C. khmerensis*, *C. metapsilosis*, *C. parapsilosis*, *C. tropicalis*, *C. glabrata*	[7,61,100,101,105,106,107,108]
*Aspergillus*	*Aspergillus* sp.	100% [106]; 100% [105]; 75% [100]; 45% [101]; 35% [7]	7	*A. amstelodami*, *A. caesiellus*, *A. flavus*, *A. oryzae*, *A. penicillioides*, *A. ruber*, *A. niger*	[7,61,100,101,105,106,107,108]
*Penicillium*	*Penicillium* sp.	97% [105]; 85% [100]; 70% [101]; 50% [106]	4	*P. brevicompactum*, *P. glabrum*, *P. spinulosum*, *P. citrinum*	[7,61,100,101,105,107]
*Cladosporium*	*Cladosporium* sp.	100% [106]; 72.5% [100]; 65% [7]	4	*C. cladosporioides*, *C. herbarum*, *C. sphaerospermu*, *C. teniussimum*	[7,61,100,106,107,108]
*Phoma*	*Phoma* sp.	100% [106]; 65% [101]	3	*P. foveata*, *P. plurivora*, *P. herbarum*	[7,61,101,106]
*Malassezia*	*Malassezia sp.*	100% [106]; 40% [101]	3	*M. restricta*, *M. globosa*, *M. sympodialis*	[106,107,108]
*Cryptococcus*	*Cryptococcus* sp.	100% [106]; 20% [7]	2	*C. cellulolyticus*, *C. diffluens*	[7,106]
*Alternaria*	*Alternaria* sp.	100% [106]; 5% [100]	4	*A. tenuissima*, *A. triticina*, *A. alternata*, *A. armoraciae*	[7,100,101,106,107,108]
*Fusarium*	*Fusarium* sp.	83% [106]; 30% [7]	3	*F. oxysporum*, *F. culmorum*, *F. poae*	[7,107,108]
*Rhodotorula*	*Rhodotorula* sp.	75% [100]	1	*R. mucilaginosa*	[100,107,108]
*Aureobasidium*	*Aureobasidium* sp.	67% [106]; 50% [7]	0	not classified	[7,100,108]
*Saccharomyces*	*Saccharomyces* sp.	50% [7]; 50% [106]; 45% [101]	3	*S. bayanus*, *S. cerevisiae*, *S. ellipsoideus*	[7,61,101,107]
*Trichoderma*	*Trichoderma* sp.	50% [106]; 45% [101]; 10% [100]	1	*T. aureoviride*	[100,101,107]

**Table 6:** The list of chosen skin mycobiome colonizers. The table summarizes the relative abundance (or frequency) of representative taxa, number and summary of all species described in the studies.

Site	Genus	The Most Common Species	Abundance/ Frequency	Number of Species	List of All Species	References
Scalp	*Malassezia*	*M sympodialis*	23.8–57.1% [125]	7	*M. sympodialis*, *M. furfur*, *M. globosa*, *M. restricta*, *M. sympodialis*, *M. dermatis*, *M. slooffiae*	[125,126]
	*Candida*	*C. albicans*	50–65% [126]	2	*C. albicans*, *C. metapsilosis*	[125,126]
	*Aspergillus*	*Aspergillus* sp.	not found	0	not classified	[125]
	*Cryptococcus*	*Cryptococcus* sp.	not found	0	not classified	[126]
	*Aureobasidium*	*Aureobasidium* sp.	not found	0	not classified	[125]
	*Acremonium*	*Acremonium* sp.	not found	0	not classified	[126]
	*Cladosporium*	*Cladosporium* sp.	not found	0	not classified	[125]
	*Hortaea*	*Hortaea* sp.	not found	0	not classified	[125]
	*Trichosporon*	*Trichosporon* sp.	not found	0	not classified	[125]
	*Pallidocercospora*	*Pallidocercospora* sp.	not found	0	not classified	[125]
	*Didymella*	*Didymella* sp.	not found	0	not classified	[126]
Areas with a high density of sebaceous glands	*Malassezia*	*M. globosa*	91% [127] 13.5–56.8% [125]	6	*M. globosa*, *M. furfur*, *M. restricta*, *M. sympodialis*, *M. dermatis*, *M. slooffiae*	[125,127]
	*Candida*	*C. parapsilosis*	6.25–35.1% [125]	2	*C. parapsilosis*, *C. orthopsilosis*	[125]
	*Alternaria*	*Alternaria* sp.	not found	0	not classified	[120]
	*Cladosporium*	*Cladosporium* sp.	not found	0	not classified	[120]
	*Cryptococcus*	*Cryptococcus* sp.	not found	0	not classified	[120]
	*Schizophillum*	*Schizophillum* sp.	not found	0	not classified	[120]
	*Rhodotorula*	*R. mucilaginosa*	13.5% [120]; 0.62% [125]	1	*R. mucilaginosa*	[120,125]
	*Penicillium*	*Penicillium* sp.	not found	0	not classified	[125]
	*Lodderomyces*	*L. elongisporus*	not found	1	*L. elongisporus*	[125]
	*Rhodosporium*	*R. toloroides*	not found	1	*R. toloroides*	[125]
	*Naganisha*	*N. albidosimilis*	not found	1	*N. albidosimilis*	[125]
Areas with a low density of sebaceous glands	*Malassezia*	*Malassezia* sp.	24.48–43.43% [120]	6	*M. globosa M. restricta*, *M. furfur*, *M. obtuse*, *M. sympodialis*, *M. japonica*	[120]
	*Mucor*	*Mucor* sp.	not found	0	not classified	[127]
	*Neurospora*	*Neurospora* sp.	not found	0	not classified	[127]
	*Aspergillus*	*Aspergillus* sp.	not found	0	not classified	[127]
	*Preussia*	*Preussia* sp.	not found	0	not classified	[127]
	*Saccharomyces*	*Saccharomyces* sp.	not found	0	not classified	[127]
	*Rasamsonia*	*Rasamsonia* sp.	not found	0	not classified	[120]
	*Alternaria*	*Alternaria* sp.	not found	0	not classified	[120]
	*Cladosporium*	*Cladosporium* sp.	5.71–9.85% [120]	0	not classified	[120]
	*Candida*	*Candida* sp.	4.42–7.05% [120]	0	not classified	[120]
	*Cryptococcus*	*Cryptococcus* sp.	not found	0	not classified	[120]
	*Rhodotorula*	*Rhodotorula* sp.	not found	0	not classified	[120]
	*Penicillium*	*Penicillium* sp.	not found	0	not classified	[120]

**Table 7:** The list of chosen genitourinary tract mycobiome colonizers. The table summarizes the relative abundance (or frequency) of representative taxa, number and summary of all species described in the studies.

Site	Genus	The Most Common Species	Abundance/ Frequency	Number of Species	List of all Species	References
Urinary tract	*Candida*	*Candida* sp.	not found	1	not classified	[145,147]
	*Saccharomyces*	*Saccharomyces* sp.	not found	1	not classified	[145]
	*Malassezia*	*Malassezia* sp.	not found	1	not classified	[145]
Vagina	*Candida*	*Candida* sp.	1–69% [148]; 36.9% [8]	9	*C. albicans*, *C. glabrata*, *C. krusei*, *C. tropicalis*, *C. parapsilosis*, *C. dubliniensis*, *C. guillermondi*, *C. kefyr*, *C. humicolus*,	[8,148]
		*C. albicans*	34.1 [8]			
	*Saccharomyces*	*S. cerevisiae*	5% [148]	1	*S. cerevisiae*	[148,149]
	*Cladosporium*	*C. perangustum*	>1% [8]	1	*C. perangustum*	[8]
	*Alternaria*	*A. alternata*	>1% [8]	1	*A. alternata*	[8]
	*Rhodotorula*	*Rhodotorula* sp.	>1% [8]	0	not classified	[8]

**Table 8:** The list of chosen respiratory tract mycobiome colonizers. The table summarizes representative taxa, number and summary of all species described in the studies.

Site	Genus	The Most Common Species	Number of Species	List of Species	References
Lungs	*Cladosporium*	*Cladosporium* sp.	0	not classified	[156]
	*Penicillium*	*Penicillium* sp.	0	not classified	[156]
	*Aspergillus*	*Aspergillus* sp.	0	not classified	[156]
	*Candida*	*Candida* sp.	0	not classified	[156]
	*Malassezia*	*Malassezia* sp.	0	not classified	[156]
	*Kluyveromyces*	*Kluyveromyces* sp.	0	not classified	[156]
	*Pneumocystis*	*Pneumocystis* sp.	0	not classified	[126,156]
Bronchoalveolar lavage	*Cladosporium*	*Cladosporium* sp.	0	not classified	[145]
	*Candida*	*C. guilliermondii*	2	*C. guilliermondii*, *C. albicans*	[20,156]
	*Aspergillus*	*Aspergillus* sp.	2	*A. fumigatus, A. flavus*	[145,156]
	*Penicillium*	*Penicillium* sp.	0	not classified	[145]
	*Saccharomyces*	*S. cerevisiae*	0	not classified	[20]
	*Pichia*	*Pichia* sp.	2	*P. Jadini*, *P. brevicompactum*	[20]
Exhaled breath Condensate (EBC)	*Cladosporium*	*Cladosporium* sp.	1	*C. herbarum*	[159]
	*Aspergillus*	*A. sydowii*	1	*A. sydowii*	[159]
	*Penicillium*	*P. brevicompactum*	5	*P. brevicompactum*, *P. Expansum*, *P. glabrum*, *P. olsonii*, *P. bilaiae*	[159]
	*Alternaria*	*A. alternata*	2	*A. alternata*, *A. infectoria*	[159]
Sputum	*Saccharomyces*	*Saccharomyces* sp.	0	not classified	[162]
	*Candida*	*Candida* sp.	2	*C. albicans*, *C. dubliniensis*	[162]
	*Ganoderma*	*Ganoderma* sp.	0	not classified	[20]
	*Malassezia*	*Malassezia* sp.	0	not classified	[162]
	*Aspergillus*	*A. fumigatus*	1	*A. fumigatus*	[20]
	*Cryptococcus*	*C. magnus*	1	*C. magnus*	[20]
	*Peniophora*	*P. incarnata*	2	*P. Incarnata*, *P. Cinerea*	[20]
	*Daedaleopsis*	*D. confragosa*	1	*D. confragosa*	[20]
	*Sistotrema*	*S. brinkmannii*	1	*S. brinkmannii*	[20]
	*Stereum*	*S. hirsutum*	1	*S. hirsutum*	[20]

## Data Availability

Not applicable.
